# Differential impacts of shared parasites on fitness components among competing hosts

**DOI:** 10.1002/ece3.3062

**Published:** 2017-05-22

**Authors:** Olwyn C. Friesen, Robert Poulin, Clément Lagrue

**Affiliations:** ^1^ Department of ZoologyUniversity of OtagoDunedinNew Zealand

**Keywords:** host behavior, host fitness, multispecies infection, parasites, survival

## Abstract

Effects of parasites on individual hosts can eventually translate to impacts on host communities. In particular, parasitism can differentially affect host fitness among sympatric and interacting host species. We examined whether the impact of shared parasites varied among host species within the same community. Specifically, we looked at the impacts of the acanthocephalan *Acanthocephalus galaxii*, the trematodes *Coitocaecum parvum* and *Maritrema poulini*, and the nematode *Hedruris spinigera*, on three host species: the amphipods, *Paracalliope fluviatilis* and *Paracorophium excavatum*, and the isopod, *Austridotea annectens*. We assessed parasite infection levels in the three host species and tested for effects on host survival, behavior, probability of pairing, and fecundity. *Maritrema poulini* and *C. parvum* were most abundant in *P. excavatum* but had no effect on its survival, whereas they negatively affected the survival of *P. fluviatilis*, the other amphipod. Female amphipods carrying young had higher *M. poulini* and *C. parvum* abundance than those without, yet the number of young carried was not linked to parasite abundance. Behavior of the isopod *A. annectens* was affected by *M. poulini* infection; more heavily infected individuals were more active. *Paracorophium excavatum* moved longer distances when abundance of *C. parvum* was lower, yet no relationship existed with respect to infection by both *M. poulini* and *C. parvum*. The differential effects of parasites on amphipods and isopods may lead to community‐wide effects. Understanding the consequences of parasitic infection and differences among host species is key to gaining greater insight into the role of parasite mediation in ecosystem dynamics.

## 1 INTRODUCTION

Community structure and dynamics are affected by the direct interactions of competition and predation, as well as indirect interactions, such as trophic cascades, keystone predation, and parasite mediation (Hatcher & Dunn, 2011; Holt & Pickering, 1985; Park, 1948; Price et al., 1986). Host responses to parasitism can vary widely and affect growth, behavior, reproduction, aging, and ability to respond to stressful conditions (Bedhomme, Agnew, Vital, Sidobre, & Michalakis, 2005; Brown & Pascoe, 1989; Cox, 2001; Thomas, Guegan, & Renaud, 2009; Thompson, Redak, & Wang, 2001). The extent of these impacts can also vary greatly among hosts, both inter‐ and intraspecifically (Shaw & Dobson, 1995). These variations among and within host species may affect competition and predation dynamics, eventually impacting the structure of the entire community (Rauque, Paterson, Poulin, & Tompkins, 2011; Smith, Acevedo‐Whitehouse, & Pedersen, 2009; Tompkins, Dunn, Smith, & Telfer, 2011).

Direct effects of parasitism on hosts can include alteration of feeding rates (Rivero & Ferguson, 2003), behavior (Lefèvre et al., 2009; Poulin, 1995; Thomas, Renaud, Demee, & Poulin, 1998), stress response (Bedhomme et al., 2005; Brown & Pascoe, 1989), survival (Lehmann, 1992), and ability to compete for resources (Park, 1948; Price, Westoby, & Rice, 1988). Parasite can also have direct effects on reproduction, through the total or partial castration of the host via gonad destruction or reduced energy stores needed for egg production (Rauque et al., 2011), or indirect ones through a reduction in pairing success or parental care (Bollache, Gambade, & Cézilly, 2001; Lefebvre, Fredensborg, Armstrong, Hansen, & Poulin, 2005; Rauque et al., 2011; Read, 1988). Changes in host behavior, even subtle, can in turn have community‐wide repercussions (Lefèvre et al., 2009; Poulin, 1995; Thomas et al., 1998). Behavioral modification can include changes in activity levels (Kunz & Pung, 2004; Leung & Poulin, 2006; Webster, 1994), position in the water column (Hansen & Poulin, 2005; Rauque et al., 2011), aggression (Mikheev, Pasternak, Taskinen, & Valtonen, 2010), boldness (Reisinger, Petersen, Hing, Davila, & Lodge, 2015), and photophilia (Bauer, Trouvé, Grégoire, Bollache, & Cézilly, 2000; Rauque et al., 2011). Alterations of host behavior may lead to increased vulnerability to predation (Kunz & Pung, 2004) and reduced ability to compete for resources (Mikheev et al., 2010; Reisinger et al., 2015). Consequently, parasites may influence the outcome of competition among hosts and impact community dynamics.

Furthermore, different parasite species can vary greatly in their impacts on hosts. For instance, the pairing success of male *Gammarus pulex* amphipods was affected differently by infection with *Pomphorhynchus laevis* than *Polymorphus minutus*, both acanthocephalan parasites (Bollache et al., 2001). Many parasite species are known to infect a variety of hosts, many of which may compete strongly with each other within their ecosystem. Differential impacts of parasites on competitors will affect their relative competitive abilities (Price et al., 1986). As species vary in their susceptibility and tolerance to parasites, the presence or absence of parasite species may dictate the coexistence of species or the complete absence of a species within an ecosystem (Greenman & Hudson, 2000; Hatcher, Dick, & Dunn, 2006). If two species are equal competitors, the presence of a parasite that infects only one of them may change this interaction. If the host is negatively affected by the parasite, it may give the competitor the advantage. The more tolerant host may also be able to act as a reservoir for the parasites, maintaining a high level of parasitism within the system (Arneberg, Skorping, Grenfell, & Read, 1998). To better understand the outcomes of competition and predation in the presence of shared parasites, it is important to understand the different impacts parasites may have on particular hosts.

Impacts of parasites on their hosts are usually studied in simple one parasite — one host species context. However, multispecies infections are not uncommon (Alizon, de Roode, & Michalakis, 2013; Hughes & Boomsma, 2004; Lagrue & Poulin, 2008a; Lange, Reuter, Ebert, Muylaert, & Decaestecker, 2014; Pedersen & Fenton, 2007; Thumbi et al., 2014). Furthermore, although each infection event is often independent, the presence of multiple parasite species within a host may have synergistic or antagonistic effects compared to the presence of one parasite alone (Alizon et al., 2013; Lagrue & Poulin, 2008a; Lange et al., 2014). However, the effects of diverse within‐host parasite assemblages can often be very difficult to predict (Alizon, 2013). Interactions among parasites may affect their respective virulence and the survival of the host (Alizon, 2013; Balmer, Stearns, Schötzau, & Brun, 2009; Lange et al., 2014; de Roode, Culleton, Cheesman, Carter, & Read, 2004). The overall virulence of a combination of parasites can be higher than that of the most virulent parasite, lower than the least virulent one or reach some intermediate level (Alizon et al., 2013). Yet, despite the importance of interactions among shared parasites and multispecies infections, little is understood about how shared parasites may shape host communities.

The objectives of our study were to examine potential impacts of different parasite species on three species of hosts in the same community. We examined the effects of parasites on invertebrates used as intermediate hosts by four parasite species, all of which are transmitted trophically to their definitive host. Parasite effects on host fecundity, behavior, and survival have been previously examined in one of the host species, the amphipod *Paracalliope fluviatilis* (Lagrue & Poulin, 2008a; Rauque et al., 2011). It serves as host to two trematode species, *Coitocaecum parvum* and *Maritrema poulini*, and the acanthocephalan *Acanthocephalus galaxii*. *Coitocaecum parvum,* and *A. galaxii* use fish as definitive hosts while *M. poulini* is an avian parasite (Hine, 1977; MacFarlane, 1939; Presswell, Blasco‐Costa, & Kostadinova, 2014). Less is known about the impacts of these parasites on other hosts in the community. Additionally, potential effects of multiple infections are not well understood. Two other crustacean species are commonly found coexisting with *P. fluviatilis* and serve as hosts to some of the same parasites. *Paracorophium excavatum*, another amphipod, is larger than *P. fluviatilis* but they both occur in sympatry (Ruiz‐Daniels, Beltran, Poulin, & Lagrue, 2012). *Paracorophium excavatum* is also host to three parasites, including the trematodes *C. parvum* and *M. poulini*, but also the fish nematode *Hedruris spinigera* (Lagrue & Poulin, 2008b; Luque, Bannock, Lagrue, & Poulin, 2007; Luque et al., 2010; Ruiz‐Daniels et al., 2012). The prevalence and abundance of *C. parvum* and *M. poulini* have previously been found to be higher in *P. excavatum* than in *P. fluviatilis* (Ruiz‐Daniels et al., 2012). The isopod *Austridotea annectens* is also found in the same area and is an intermediate host for *M. poulini* (Hansen & Poulin, 2005; Presswell et al., 2014). Many of these parasites reach a relatively large size and/or abundance within their hosts, suggesting potential impacts on host survival and behavior (Rauque et al., 2011). Our specific objectives were to (i) determine whether parasite effects varied among hosts within the same community and (ii) test whether multispecies infections had synergistic or antagonistic effects compared to the presence of single parasite infection (Alizon et al., 2013; Lagrue & Poulin, 2008a; Lange et al., 2014). As these hosts are all competing for resources and share a variety of parasites, a better understanding of the impacts of parasitism on each host is necessary to understand how parasites affect population dynamics in this community.

## 2 METHODS

### 2.1 Sample collection

We collected samples of naturally infected amphipods and isopods from the littoral zone of Lake Waihola, South Island, New Zealand (46°01′14S, 170°05′05E) between February and September 2016. Sampling for survival tests occurred over 3 days, 9 February, 21 March, and 1 May 2016. Sampling for the behavioral tests occurred over 3 days, 13 May, 1 June, and 6 September 2016, due to seasonal variation in host abundance. Sampling for pairing behavior occurred on 21 March 2016 for *P. fluviatilis* (as described below) but paired isopods were collected during the entire sampling period as they occurred far less frequently. Data on the size, sex, and the prevalence and abundance of parasites in each host species from each sample event were pooled for examination of intraspecific and interspecific variation. Animals were caught using dip‐nets and transported to the laboratory in lake water. Amphipods and isopods were transferred and maintained separately by species in 10 L tanks containing aerated lake water. Animals were kept at room temperature (14 ± 1°C) with aquatic plants (*Myriophyllum triphyllum* and *Elodea canadensis*) for food, and under a controlled photoperiod (12‐hr dark and light).

All amphipods used in behavioral and fecundity trials were subsequently dissected within a week of collection as keeping these amphipods in the laboratory for long periods of time can affect amphipod survival (Lagrue & Poulin, 2007; Lagrue, Poulin, & Keeney, 2009; Poulin, 2003). All isopods used in behavioral trials were dissected within a month of collection. If individuals did not die during trial, they were killed in 70% ethanol and rinsed in distilled water before dissection. In our study, prevalence was defined as the percentage of infected hosts, abundance was defined as the number of parasites per host including zeroes, and mean abundance as the mean number of parasites among a specific sample of hosts.

### 2.2 Survival tests

Within 6 hr after sampling, 266 *P. fluviatilis*, 210 *P. excavatum,* and 390 *A. annectens* were separated into individual wells of tissue culture plates. According to host size, *P. fluviatilis* were maintained in 96‐well microplates with 300 μL of water per well, *P. excavatum* were maintained in 24‐well plates with 500 μL of water per well, and *A. annectens* were kept in 12‐well plates with 1 mL of water per well. All individuals were maintained at the same temperature (14 ± 1°C) and photoperiod (12‐hr dark and light) but no food was added. Well plates were checked daily for any dead individuals. If a female released young, the number of young was recorded and they were removed from the well as they could have provided an additional food resource to the focal animal through cannibalism. If individuals could be dissected the same day, they were left in lake water. For dissection occurring more than 24 hr after death, the individuals were immediately preserved in 70% ethanol until dissection. The total body length of each individual was determined by measuring from the anterior tip of the cephalic capsule to the posterior end of the uropods. Sex was determined for each individual when possible. Isopods shorter than 7–8 mm in body length were impossible to sex due to the lack of secondary sexual characters and thus considered juveniles. Egg presence and number were also recorded. Individuals were then dissected to identify and count parasites.

### 2.3 Behavioral tests

Amphipods and isopods were individually isolated (in wells of culture plates, as described above) within 6 hr of sampling and left for 12 hr to acclimate to their new environments. Individuals were subsequently filmed to record velocity and activity levels. Infection status of each individual was unknown during filming and subsequent video analysis. Fine sand was added to the wells of *P. excavatum* and *A. annectens* to simulate natural conditions as both species are benthic and may use sand to burrow. One hour prior to recording, plates were moved to the filming studio to allow the animals to adjust to the temperature (18 ± 1°C) and lighting changes. *Paracalliope fluviatilis* and *P. excavatum* were filmed for 5 min under a dissecting microscope (Olympus SZ61, 0.65× magnification) due to their small size. Well plates containing *A. annectens* were filmed for 5 min using a Canon digital camera (1200D). Activity levels (distance moved from center (mm), mean velocity (mm/ms), highly mobile duration (more than 60% of the animal (measured by pixels altered) has moved within the sample period (0.05 s), which is then calculated as a proportion of the entire sample to give a measure of high‐speed movement), and mobile duration (more than 20% of the animal (measured by pixels altered) has moved within the sample period (0.05 s), which is then calculated as a proportion of the entire sample to give a measure of movement)) were calculated for each individual over 5 min using EthoVision XT (Noldus Information Technology 2015). Male and female amphipods were combined as no difference in behavior between sexes was observed (*P. fluviatilis*: distance moved from center, Kruskal–Wallis tests *Z* = 1.3, *p *=* *.19; mean velocity, Kruskal–Wallis tests *Z* = 1.3, *p *=* *.19; highly mobile duration, Kruskal–Wallis tests *Z* = 1.2, *p *=* *.23; and mobile duration, Kruskal–Wallis tests *Z* = −0.93, *p *=* *.35; *P. excavatum*: distance moved from center, Kruskal–Wallis tests *Z* = −0.42, *p *=* *.67; mean velocity, Kruskal–Wallis tests *Z* = −0.40, *p *=* *.68; highly mobile duration, Kruskal–Wallis tests *Z* = 0.58, *p *=* *.58; and mobile duration, Kruskal–Wallis tests *Z* = 0.014, *p *=* *.99).

### 2.4 Fecundity and pairing probability

Offspring carried in the brood pouch of gravid females (from the survival and behavioral tests) were counted and recorded with their corresponding body length and parasite burdens. If any young were released during survival tests, the number of young was recorded and matched with the female's corresponding parasite burden upon death. The parasite burden of females without any young was also compared to those with young to examine whether a relationship existed between the probability of having young and a female's parasite burden.

Additionally, a subset of paired *P. fluviatilis* and *A. annectens* were identified and individually separated into tissue culture microtest tubes within 12 hr of capture. The paired individuals consisted of a male clasping a female in a precopulatory pair. Nonpaired individuals were also collected during the same sampling event and separated into individual tubes. Amphipods were dissected as described above within 24 hr of capture. Isopods were euthanized and preserved in ethanol and dissected as described above.

### 2.5 Statistical analysis

Statistical analyses were performed in JMP^®^ 12 (SAS Institute Inc 2015) and R statistical software (http://www.R-project.org). Size differences between sexes were examined using a Kruskal–Wallis test. The relationship between host size and parasite abundance was assessed using Spearman's correlations. Differences in parasite abundance and prevalence among host species and between sexes of each individual parasite species were examined using a Kruskal–Wallis test and contingency analysis, respectively. The relationship between either survival (days before death) or behavioral measures, and parasite abundance of *C. parvum*,* M. poulini*,* A. galaxii*, and *H. spinigera*, as well as multispecies combinations, was analyzed separately for each host species using a negative binomial regression, with host size being included as an additional explanatory variable and their interactions when significant. Logistic regression was used to relate parasite abundance with both the likelihood of being paired in *P. fluviatilis* and *A. annectens* by sex and the probability of having young in female *P. fluviatilis* and *P. excavatum*. The number of young carried by a female was related to its body size and parasite abundance using negative binomial regression.

## 3 RESULTS

Parasite prevalence and abundance varied greatly among host species (Table 1). The highest number of individual of *M. poulini* per host was 74 (in *A. annectens*), 8 for *C. parvum* (in *P. excavatum*), and 1 for both *A. galaxii* (in *P. fluviatilis*) and *H. spinigera* (in *P. excavatum*). Multiple infections were found in both amphipod species, with *C. parvum* and *M. poulini* co‐infecting 1.65% of *P. fluviatilis* and 20.7% of *P. excavatum*, and *M. poulini* and *H. spinigera* co‐infecting 3.7% of *P. excavatum*. One *P. excavatum* (0.34%) was infected with three parasite species (*C. parvum, M. poulini,* and *H. spinigera*). *Acanthocephalus* *galaxii* was not found sharing the same individual host with any other parasites within our sample. The abundance and prevalence of *M. poulini* varied significantly among all hosts (abundance: Kruskal–Wallis tests, χ^2^ = 967, *p *<* *.0001; prevalence: Contingency analysis, χ^2^ = 1033.6, *p *<* *.0001), with the abundance being highest in *P. excavatum* and lowest in *P. fluviatilis* (Table 1). A synopsis of the key results of this study is summarized in Table 2.

**Table 1:** Parasite prevalence and abundance (mean ± *SE*) in the three host species (*Paracalliope fluviatilis*,* Paracorophium excavatum*, and *Austridotea annectens*). All samples were collected from Lake Waihola, South Island, New Zealand between February and September 2016

Species	Sex	*N*	Size (mm)	Prevalence (%)					Abundance				
				*AG*	*CP*	*HS*	*MP*	Total	*AG*	*CP*	*HS*	*MP*	Total
*Paracalliope fluviatilis*	Female	687	2.0 ± 0.021	0.9	19	–	5.8	24	0.009 ± 0.004	0.26 ± 0.026	–	0.084 ± 0.015	0.35 ± 0.030
	Male	220	2.6 ± 0.036	3.3	23	–	12	34	0.032 ± 0.012	0.29 ± 0.041	–	0.20 ± 0.043	0.52 ± 0.061
*Paracorophium excavatum*	Female	211	4.2 ± 0.040	–	24	3.3	91	92	–	0.39 ± 0.065	0.033 ± 0.012	12.8 ± 0.79	13 ± 0.79
	Male	83	4.6 ± 0.47	–	16	6.0	84	89	–	0.22 ± 0.062	0.060 ± 0.026	9.7 ± 1.2	9.9 ± 1.2
*Austridotea annectens*	Female	21	8.4 ± 0.18	–	–	–	86	86	–	–	–	14 ± 3.6	14 ± 3.6
	Male	24	8.8 ± 0.29	–	–	–	88	88	–	–	–	27 ± 4.0	27 ± 4.0
	Juvenile	399	5.2 ± 0.049	–	–	–	71	71	–	–	–	5.2 ± 0.30	5.2 ± 0.30

*AG*,* Acanthocephalus galaxii; CP*,* Coitocaecum parvum*;* HS*,* Hedruris spinigera; MP*,* Maritrema poulini*.

**Table 2:** Summary of infection levels and key effects of parasites on all three hosts. All samples were collected from Lake Waihola, South Island, New Zealand between February and September 2016. Significant effects are shown in bold

Hosts		Parasites					
		*A. galaxii* (acanthocephalan)	*C. parvum* (trematode)	*H. spinigera* (nematode)	*M. poulini* (trematode)	Multiple infection (*M. poulini* and *C. parvum)*	Multiple infection (*M. poulini* and *H. spinigera)*
*Paracalliope fluviatilis* (amphipod)	Prevalence	Low	Low	–	Low	Low	–
	Abundance	Low	Low		Low	Low	
	Survival	No effect	**Reduced**		**Reduced**	**Reduced**	
	Activity	**Reduced**	No effect		No effect	No effect	
	Fecundity	No effect	**More with eggs**		**More with eggs**	No effect	
	Pairing ‐ ♀	**Higher**	**Lower**		No effect	No effect	
	♂	No effect	No effect		No effect	No effect	
*Paracorophium excavatum* (amphipod)	Prevalence	–	Low	Low	High	Low	Low
	Abundance		Low	Low	High	Low	Low
	Survival		No effect	No effect	No effect	No effect	No effect
	Activity		**Reduced**	No effect	No effect	No effect	No effect
	Fecundity		**More with eggs**	No effect	**More with eggs**	No effect	Fewer with eggs
	Pairing ‐ ♀		–	–	–	–	–
	♂						
*Austridotea annectens* (isopod)	Prevalence	–	–	–	High	–	–
	Abundance				High		
	Survival				**Improved**		
	Activity				**Increase**		
	Fecundity				–		
	Pairing ‐ ♀				No effect		
	♂				Higher		

Amphipod sex ratio was approximately 3:1 females to males for *P. fluviatilis* and 2.5:1 females to males for *P. excavatum*. Isopod sex ratio was approximately 1:1 among individuals that were large enough to be sexed. Size differences between sexes were found in *P. fluviatilis* (Kruskal–Wallis test, *Z* = 12.68, *p *<* *.001) but not *P. excavatum (Z* = −0.47, *p *=* *.64), or *A. annectens* (*Z* = −1.62, *p *=* *.10). Sex and size were both related to parasite infection in *P. fluviatilis*. As observed in previous studies, abundance and prevalence of *M. poulini* (Kruskal–Wallis test, *Z* = 3.1, *p *= .002; contingency analysis, χ^2^ = 8.9, *p *=* *.003) and *A. galaxii* (Kruskal–Wallis test, *Z* = 6.3, *p *= .012; contingency analysis, χ^2^ = 5.3, *p *= .021) were higher in male than female *P. fluviatilis* (Rauque et al., 2011). However, no difference was found between males and females of *P. fluviatilis* in the abundance or prevalence of *C. parvum* (all *p*‐values >.05). *Paracalliope fluviatilis* size was positively related to *C. parvum* abundance (Spearman ρ = 0.10, *p *=* *.0025) and multiple infection of *C. parvum* and *M. poulini* (Spearman ρ = 0.078, *p *=* *.019) but not related to *A. galaxii* or *M. poulini* abundance (all *p*‐values >.05). Analyzed separately, the size of female *P. fluviatilis* was positively related to *C. parvum* abundance (Spearman's ρ = 0.11, *p *=* *.0044) but male size was not (Spearman's ρ = 0.03, *p *=* *.63). Separating the sexes did not change the lack of relationship between size and the abundance of *A. galaxii* and *M. poulini* for females or males (all *p*‐values >.05).

In contrast, female *P. excavatum* had a higher abundance and prevalence of *M. poulini* (Kruskal–Wallis test, *Z* = −2.24, *p *= .025; contingency analysis, χ^2^ = 5.76, *p *= .016) and *C. parvum* (Kruskal–Wallis test, *Z* = −1.70, *p *= .089; contingency analysis, χ^2^ = 2.59, *p *= .11) than males. *Paracorophium excavatum* size was positively related to *M. poulini* abundance (Spearman's ρ = 0.19, *p *=* *.0014). However, size was not related to *H. spinigera* abundance, *C. parvum* abundance, or multiple infections (all *p*‐values >.05). Analyzing the sexes separately, both male and female *P. excavatum* size, is related to *M. poulini* abundance (females, Spearman's ρ = 0.41, *p *<* *.001; males, Spearman's ρ = 0.25, *p* = .021).

Isopods were infected by *M. poulini* only. The abundance of *M. poulini* in *A. annectens* varied greatly between sexes, with males having the highest abundance, followed by females, and then juveniles (ANOVA, F_2,441_=95.24, *p *<* *.001, Tukey's HSD post hoc test, all *p* < .001). Size was positively related to *M. poulini* abundance (Spearman's ρ = 0.33, *p *<* *.001).

### 3.1 Host survival and parasite infection

**Figure 1:**
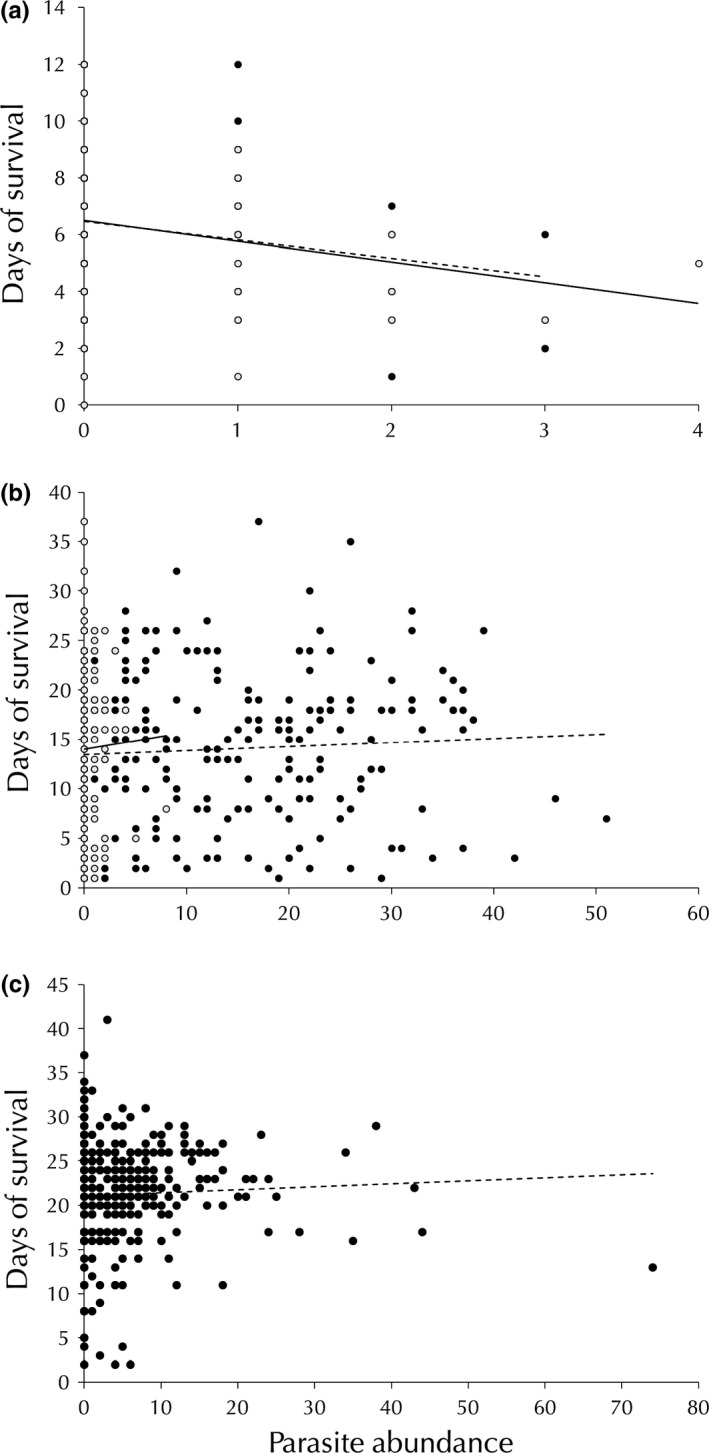
Relationship between *Coitocaecum parvum* (○, solid line) and *Maritrema poulini* (●, dashed line) abundance and days of survival in all hosts, (a) *Paracalliope fluviatilis* (*n* = 266; *C. parvum* abundance: negative binomial regression, *z* = −2.44, *p *=* *.015; *M. poulini* abundance*: z = *−2.08*, p *=* *.038), (b) *Paracorophium excavatum* (*n* = 210; *C. parvum* abundance: *z* = 0.32, *p *=* *.75; *M. poulini* abundance*: z = *0.78*, p *=* *.44), and (c) *Austridotea annectens* (*n* = 390; *M. poulini* abundance*: z = *3.66*, p *=* *.0003). Regression lines represent the direction of relationships. All animals were collected from Lake Waihola, New Zealand between February and September 2016

The survival (i.e., number of days before death) of *P. fluviatilis* was negatively related to *M*. *poulini* and *C. parvum* abundance and host size (Table 4, Figure 1). There was no interactive effect between either parasite abundance and size (Table 4). Survival was not related to the abundance of *A. galaxii*. However, there was an interactive effect between host size and *A. galaxii* abundance (Table 4).

No relationship was found between host survival and *M*. *poulini*,* C. parvum*, or *H. spinigera* abundance in *P. excavatum* (Table 4, Figure 1). Intriguingly, there was a positive relationship between *M. poulini* abundance and survival in *A. annectens*, as well as a relationship between host size and parasite abundance with an interactive effect between size and abundance (Table 4, Figure 1).

### 3.2 Host behavior

The distance moved from the center in *P. fluviatilis* depended on the abundance of *A. galaxii* (Table 4). While all other measures of behavior in *P. fluviatilis* (velocity, high mobile duration, and mobile duration) were unaffected by parasite abundance (*A. galaxii, M. poulini,* or *C. parvum*). *Paracalliope fluviatilis* size did influence host behavior, as larger hosts moved further and had longer highly mobile durations (Table 4).

In *P. excavatum*, the distance moved from the center of the well plate was negatively related to the abundance of *C. parvum* with a significant interactive effect between host size and *C. parvum* abundance (Table 4). Additionally, individuals who spent more time highly mobile tended to have a lower abundance of *C. parvum* or to be smaller, with a trend toward an interactive effect between *C. parvum* and size (Table 4). Mobile duration (time spent moving) of *P. excavatum* was negatively related to size and tended to be positively related to *M. poulini* abundance with a trend toward an interactive effect between *M. poulini* abundance and size (Table 4). However, there was no relationship between or distance moved or high mobile duration and the abundance of *M. poulini*,* H. spinigera,* or size (Table 4). Mobile duration was not related to the abundance of *A. galaxii* or that of *C. parvum*. Velocity (mm/ms) was not related to the abundance of any parasite or size (Table 4).

Mobile duration (time spent in motion) of *A. annectens* was negatively related to *M. poulini* abundance but not to host size, although there was a significant interaction effect (Table 4, Figure 3). High mobile duration was not related to *M. poulini* abundance, but it was related to size (Table 4). No relationship was found between velocity (ms/s) or distance moved from center and *M. poulini* abundance or size (Table 4).

### 3.3 Fecundity and pairing probability

A subset of 141 *P. fluviatilis* had young (mean ± *SE*, 3.6 ± 0.16 per female). The number of young carried was not related to either *M. poulini* or *C. parvum* abundance (all *p*‐values >.05). The number of young carried was positively related to amphipod size (*Z* = 5.6, *p* > .001), with larger females carrying more young. There was no relationship between multispecies infection abundance, that is, *C. parvum* with *M. poulini*, and the number of young (*Z* = 0.065, *p *= .95).

**Figure 2:**
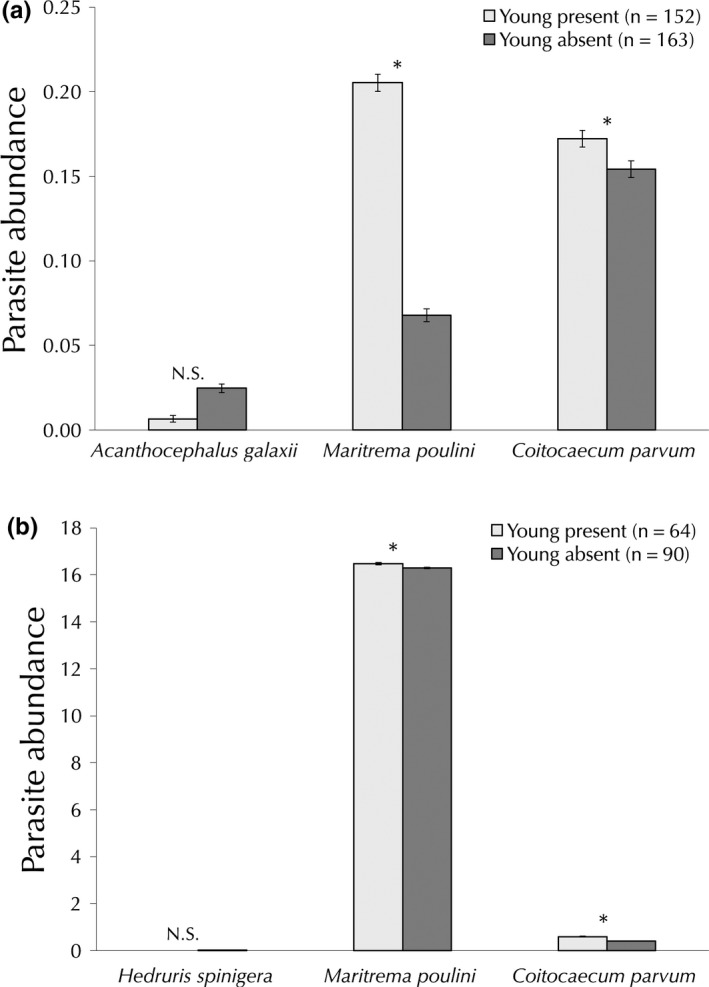
Differences in parasite abundance (mean ± *SE*) between amphipods with young (open bars) or without (filled bars) present in (a) *Paracalliope fluviatilis* and (b) *Paracorophium excavatum*. All animals were collected from Lake Waihola, New Zealand between February and September 2016. *p <* *.05, N.S. not significant for difference in abundance. Sample sizes shown inside key

We found a higher abundance of *M. poulini* (logistic regression, χ^2^ = 17.1, *p *<* *.001) and *C. parvum* (χ^2^ = 4.2, *p* = .04) in female *P. fluviatilis* with young (Figure 2). Consistently, the prevalence of *M. poulini* was higher in females with young (Contingency analysis, χ^2^ = 21, *p *<* *.0001). However, the prevalence of *C. parvum* was higher in females without young (χ^2^ = 8.8, *p *=* *.0031). There was no relationship between the presence of young and *A. galaxii* or multispecies infection abundance or prevalence (all *p*‐values >.05). There was a relationship between the size of a female *P. fluviatilis* and the likelihood of having young (χ^2^ = 38, *p *< .001), with the mean size of females with young (1.8 ± 0.024 mm) being significantly smaller than those without (2.1 ± 0.025 mm).

A subset of 71 *P. excavatum* had young (mean = 6.24 ± 0.01). The number of young *P. excavatum* carried was not related to the abundance of *H. spinigera*,* M. poulini, C. parvum*, or female size, and there was no interactive effect (all *p*‐values >.05). Females with a higher abundance of *C. parvum* (χ^2^ = 4.0, *p *= .046) and *M. poulini* (χ^2^ = 4.7, *p *= .031) were more likely to have young (Figure 2). However, we found no relationship between the abundance of *H. spinigera* and the likelihood of having young in *P. excavatum* (logistic regression, χ^2^ = 1.4, *p *= .24; Figure 2). There was no relationship between multispecies infections by *C. parvum* with *M. poulini* (χ^2^ = 1.2, *p *= .27); however, there was a trend of higher abundance of *H. spinigera* and *M. poulini* in *P. excavatum* without young than those with young (χ^2^ = 3.1, *p *= .076). We were unable to examine any relationships between the fecundity of *A. annectens* and the abundance of *M. poulini* due to a low sample size.

A sample of paired and unpaired *P. fluviatilis* (Table 3) was used to examine potential parasite effects on the likelihood of pairing in both sexes. The abundance of *A. galaxii* was higher in paired females than unpaired females (Table 3). Additionally, the prevalence of *C. parvum* was higher in nonpaired females compared to paired females (Table 3). No other differences were found in parasite abundance between paired and unpaired female and male *P. fluviatilis* (including multiple infections, Table 3).

**Table 3:** Parasite abundance (mean ± *SE*) and prevalence (percentage of sample infected) in paired and unpaired *Paracalliope fluviatilis* and *Austridotea annectens* (listed with sample size per group). All samples were collected from Lake Waihola, South Island, New Zealand between February and September 2016

Host	Parasite	Males				Females			
		Paired	Non‐paired	χ^2^	*p*	Paired	Non‐paired	χ^2^	*p*
		*n* = 64	*n* = 156			*n* = 60	*n* = 627		
*Paracalliope fluviatilis*	*A. galaxii*	0.016 ± 0.016	0.040 ± 0.016	0.88	.35	0.05 ± 0.028	0.0048 ± 0.0028	7.0	**.0083**
		1.6%	3.8%	0.58	.45	4.9%	4.8%	6.5	**.011**
	*C. parvum*	0.19 ± 0.068	0.29 ± 0.050	0.85	.36	0.15 ± 0.11	0.25 ± 0.026	1.5	.23
		17%	25%	2.1	.15	6.6%	20%	9.5	**.0021**
	*M. poulini*	0.19 ± 0.08	0.13 ± 0.044	0.79	.37	0.12 ± 0.060	0.065 ± 0.014	2.3	.13
		14%	11%	0.29	.59	9.8%	5.4%	1.3	.25
	*M. poulini + C. parvum*	0.097 ± 0.0.01	0.077 ± 0.004	0.35	.56	0.097 ± 0.013	0.040 ± 0.0009	0.74	.39
		3.1%	3.8%	0.075	.78	1.6%	1.2%	0.045	.83

		*n* = 13	*n* = 30			*n* = 11	*n* = 27		
*Austridotea annectens*	*M. poulini*	15 ± 3.6	21 ± 3.5	1.2	.28	17 ± 6.7	11 ± 3.0	0.88	.35
		100%	87%	3.1	.081*	91%	74%	1.5	.22

Significant differences through logistic regressions are shown in bold, Trends followed by *.

Paired and unpaired *A. annectens* were used to examine the possible influence of parasites on the likelihood of pairing. No difference in *M. poulini* abundance was found in either males or females between paired and unpaired individuals (Table 3). However, there was a trend for a higher prevalence of *M. poulini* in paired male isopods compared to nonpaired individuals (Table 3).

## 4 DISCUSSION

Understanding impacts of shared parasites on a variety of sympatric host species is necessary for making predictions on the potential role of parasites in ecosystem structure and functioning. We found that the impacts of parasites varied among host and parasites species within our study community. Survival rates varied among amphipods, with one showing a reduced lifespan when infected by either of the two shared parasites, whereas survival of the other amphipod species was not affected. We also found evidence that when a host species is infected by multiple parasites, the effects of each parasite may have been antagonistic to one another, with a net neutral effect on the host.

Infection levels varied among hosts within the community. *Paracorophium excavatum* had higher abundance of *M. poulini* than *P. fluviatilis* (Table 1)*,* which is consistent with previous studies (Presswell et al., 2014; Ruiz‐Daniels et al., 2012). Variation in host size or other biological characteristics may lead to these differences (Grutter & Poulin, 1998; Johnson, Bush, & Clayton, 2005; Ruiz‐Daniels et al., 2012; Saad‐Fares & Combes, 1992). *Paracorophium excavatum* is a larger amphipod that has a more benthic habitat than *P. fluviatilis* as it is often found burrowing in the sand. However, we only found a positive correlation between the size of *P. excavatum* and one species of parasite, contrary to previous studies (Presswell et al., 2014; Ruiz‐Daniels et al., 2012). The lack of difference in prevalence and abundance of *C. parvum* between amphipod species may have been due to the low overall abundance and prevalence of *C. parvum* within the community, making it more difficult to detect any effect of this parasite on its hosts. Additionally, as discussed more thoroughly below, we found no relationship between the abundance of *M. poulini* and the survival of *P. excavatum,* suggesting that interspecific differences in abundance between the two amphipods may be due to higher parasite‐induced mortality in *P. fluviatilis* following infection and/or parasite accumulation.

The impact of parasites on survival varied among the three host species. The amphipod *P. fluviatilis* was shown to have reduced survival when infected with both *C. parvum* and *M. poulini* (Table 4, Figure 1). Individuals with multispecies infections also had a lower survival than individuals with no parasites. Reduced survival incurred by hosts may be due to energetic costs of infection. It has been previously suggested that the negative effect of *M. poulini* (previously referred to as *Microphallus* sp.) on *P. fluviatilis* is a direct consequence of high parasite abundance (Rauque et al., 2011). Our results suggest that parasite‐induced mortality may influence differences in parasite abundance between amphipod species rather than size differences between the two host species. *Paracorophium excavatum* survival was not impacted by the abundance of any of its three parasites (Table 4, Figure 1). The stark contrast in response to similar parasites by the two amphipod species may be due to a difference in the virulence of the parasite between hosts as documented in several prior studies (Jensen, Thomas Jensen, & Mouritsen, 1998; Park, 1948; Thomas et al., 1995). The positive relationship between *M. poulini* abundance and survival in *A. annectens* was surprising although it may be an effect of host size (Table 4, Figure 1). The abundance of *M. poulini* in this isopod may be directly linked to exposure over time and as age is linked to size, a relationship between both is not surprising. Additionally, larger individuals may be able to survive longer, due to a higher resource base to draw upon, therefore creating the appearance of a positive relationship between parasite abundance and survival.

**Table 4:** Negative binomial regression models for relative host survival and behavior measures compared to parasite infection and host size. All samples were collected from Lake Waihola, South Island, New Zealand between February and September 2016

Species	Predictor	Survival		Behavioral measures							
				Distance moved (cm)		Velocity (cm/ms)		High mobile duration		Mobile duration	
		*z*	*p*	*z*	*p*	*z*	*p*	*z*	*p*	*z*	*p*
*Paracalliope fluviatilis*	*AG*	−1.37	.17	−2.30	**.021**	−0.24	.81	−0.002	.99	−0.74	.46
	*CP*	−2.44	**.015**	−0.49	.63	−0.16	.87	−0.76	.45	−0.28	.78
	*MP*	−2.08	**.038**	0.28	.78	0.096	.92	0.21	.83	0.017	.99
	*CP + MP*	−2.27	**.023**	0.98	.33	0.38	.71	0.95	.34	0.70	.48
	Size	0.60	.55	5.73	**<.0001**	1.36	.17	3.67	**.00025**	1.26	.21
	Size*AG	−1.98	**.048**	−0.28	.78	−0.022	.98	0.00	1.00	−0.027	.99
	Size*CP	−0.70	.48	0.63	.53	0.22	.83	−0.15	.88	1.71	.09
	Size*MP	0.30	.76	−2.19	**.029**	−0.57	.57	−1.40	.17	−0.92	.36
	Size*CP + MP	−1.26	.21	−2.19	**.029**	−0.54	.59	−1.89	.06	−1.05	.29
*Paracorophium excavatum*	*CP*	0.32	.75	−2.0	**.049**	−0.40	.69	−1.6	.10*	−1.23	.22
	*HS*	0.97	.33	0.15	.88	0.011	.99	0.0	1.0	−0.7	.48
	*MP*	0.78	.44	−0.32	.75	−0.18	.86	0.032	.97	−1.7	.097*
	*CP + MP*	0.18	.86	−0.19	.85	0.047	.96	−0.11	.91	−0.89	.37
	*MP + HS*	0.82	.41	0.15	.88	−0.044	.97	0.0	1.0	1.2	.22
	*Size*	0.44	.66	−0.54	.59	−0.31	.76	−1.8	.07*	−2.7	**.0065**
	Size*CP	−1.45	.16	1.9	**.053**	0.43	.67	1.7	.083*	1.6	.10*
	Size*HS	1.54	.12	−0.27	.79	−0.035	.97	0.0	1.0	0.69	.49
	Size*MP	1.56	.12	0.39	.70	0.22	.83	−0.0070	.99	1.8	.074*
	Size*CP + MP	−0.40	.69	0.17	.87	−0.081	.94	−0.024	.98	0.73	.47
	Size*MP + HS	1.41	.16	−0.18	.86	0.039	.97	0.0	1.0	−1.2	.22
*Austridotea annectens*	*MP*	3.66	**.0003**	0.29	.77	−0.67	.50	0.81	.42	2.88	**.004**
	Size	4.62	**<.0001**	−0.41	.68	−0.30	.76	3.70	**.00025**	−0.88	.38
	Size*MP	−3.81	**.0001**	1.40	.16	0.023	.45	0.32	.75	2.24	**.025**

*AG*,* Acanthocephalus galaxii; CP*,* Coitocaecum parvum*;* HS*,* Hedruris spinigera; MP*,* Maritrema poulini*.

Significant values are shown in bold, trends followed by *.

**Figure 3:**
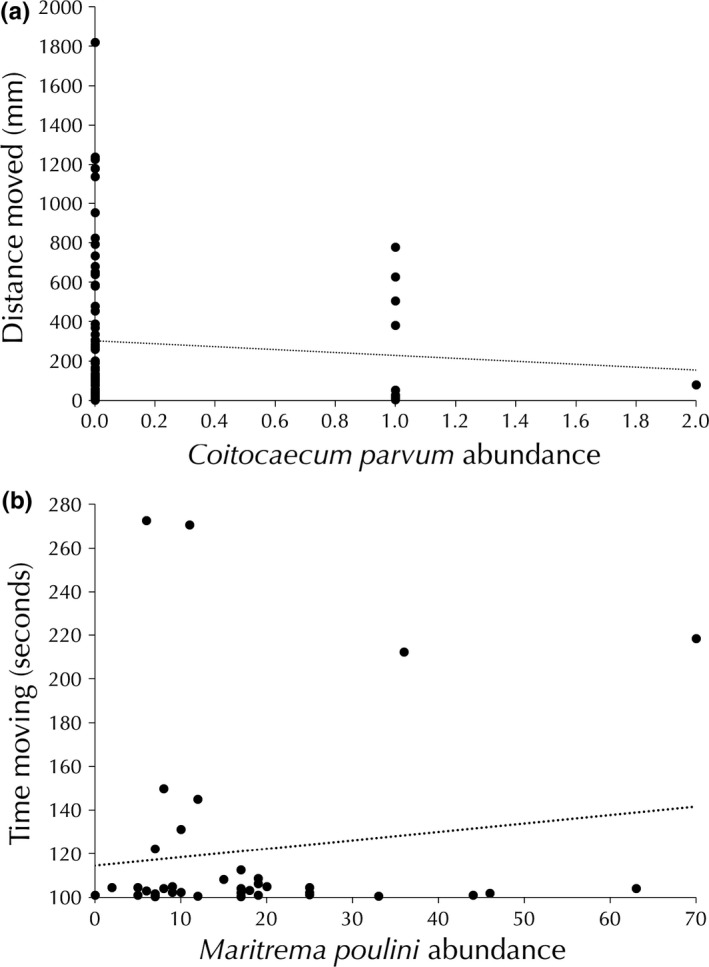
Host behavior measures compared to parasite abundance; (a) distance moved from center versus the abundance of *Coitocaecum parvum* in *Paracorophium excavatum* (*n* = 84; negative binomial regression, *z* = −2.0, *p *=* *.049) (b) mobile duration (time moving) of individual compared to their *Maritrema poulini* abundance in *Austridotea annectens* (*n* = 63; *z* = 2.88, *p *=* *.004). All measures were taken during a 5‐min behavioral observation. Regression lines represent the direction of relationships. All animals were collected from Lake Waihola, New Zealand between February and September 2016.

Hosts exhibited different behavioral responses related to the abundance of parasites. Interestingly, *P. fluviatilis* only demonstrated behavioral changes when infected with *A. galaxii*. We did not observe behavioral effects of the other parasite species as seen in a previous study (Rauque et al., 2011), and observations of parasite‐induced behavioral modification in this amphipod species have been mixed (Poulin, 2001). The inconsistency with the first study could be due to the behavioral aspects measured (phototaxis versus activity levels) (Rauque et al., 2011). In contrast, *P. excavatum* movement decreased when infected with an increasing abundance of *C. parvum* (Table 4, Figure 3). Interestingly, this relationship disappears when the host is co‐infected with *M. poulini*, suggesting an antagonistic relationship, rather than the additive effect of parasites that is often assumed. Previous studies have shown a similar trend, with photophilia increasing with *C. parvum* and *A. galaxii* infection but negated by co‐infection with *M. poulini* (Table 4), possibly due to a subtle manipulative effect being impaired by *M. poulini* (Rauque et al., 2011). The negative relationship between movement and abundance of *C. parvum* appears nonadaptive for the parasite, as increased movement can increase risk of predation, allowing *C. parvum* to be passed on to its fish definitive host, mainly the common bully (*Gobiomorphus cotidianus*). The common bully can use nonvisual methods, such as olfactory, tactile, or lateral‐line prey detection, to find prey and the lack of moment in *Paracorophium excavatum* may allow them to avoid being depredated by this fish (Rowe, 1999; Rowe, Nichols, & Kelly, 2001). The decrease in activity levels may be due to reduced energy available due to parasite infection.

Parasites had intriguing impacts on the fecundity and probability of pairing in the various hosts. Female *P. fluviatilis* and *P. excavatum* carrying young had a higher abundance of *M. poulini*. Previous studies have indicated that “handicapped’ *P. fluviatilis* females, that is, with artificially impaired swimming performance, were more likely to be paired (Sutherland, Hogg, & Waas, 2007), and therefore, females with parasites may also have been easier to pair with, explaining the higher likelihood of having young in females with a higher parasite abundance. However, the abundance of both *C. parvum* and *M. poulini* was not linked with the probability of male or female *P. fluviatilis* being found in pairs, contrary to previous studies (Rauque et al., 2011). An alternative hypothesis may suggest that females may choose to invest more into reproduction while infected; therefore, more infected female amphipods would be carrying young (Agnew, Koella, & Michalakis, 2000). Yet, we did not find any relationship between the number of young carried and the abundance of any parasites.

Multispecies infections have important implications for the fitness of individuals and the dynamics of populations. In our study community, the two amphipods had quite different levels of co‐infection, with *P. fluviatilis* quite low in comparison with *P. excavatum*. Our results suggest that these multispecies infections may be more important in *P. excavatum* as it appears that co‐infection of *M. poulini* with *C. parvum* eliminates any behavioral shift by *P. excavatum*. It has been suggested that *M. poulini* may have a greater effect on hosts than *A. galaxii* and *C. parvum* and therefore may overwhelm their subtle effects (Rauque et al., 2011). Additionally, when examining the probability of having young in female amphipods relative to parasite abundance, we found no difference when a multispecies infection of *M. poulini* and *C. parvum* was present, suggesting there may be an antagonistic relationship between parasites, cancelling out the effects of infection by either one alone. Alternatively, there were few females with young harboring multispecies infections, suggesting that if there was a difference in relationship between mixed infections and fecundity, it may be difficult to detect. Our results are consistent with other studies examining co‐infections in amphipods; for example, co‐infected *Gammarus pulex* showed a mixed response compared to individuals with single‐species infections (Cezilly, Gregoire, & Bertin, 2000). As multispecies infections are more common in *P. excavatum* than *P. fluviatilis* and appear to potentially be more important to the impacts of parasites on this host species, these co‐infection effects may have impacts on the host population overall.

Our study was based on natural infections rather than an experimental approach thus limiting our interpretations, as we cannot directly address causality or the direct mechanisms of these interactions. However, if combined with what is known of the mechanistic bases of parasite‐induced host modifications (see Introduction section), it remains a strong approach for examining the effects of parasites on their hosts (Poulin, 2001).

Interestingly, the effects of parasites on survival and behavior varied greatly between amphipod hosts. Not only does *P. excavatum* have a much higher abundance and prevalence of *M. poulini* but there appears to be no decreased survival with a high abundance of either trematode parasite (Table 2). If *P. fluviatilis* and *P. excavatum* are competing for the same resources, this may give *P. excavatum* an advantage. A more tolerant host, such as *P. excavatum,* may also be able to act as a reservoir for the parasites, maintaining a high level of parasitism within the system (Arneberg et al., 1998). This may increase infection risk for *P. fluviatilis* with possible consequences for behavior, reproduction, and/or survival. The more tolerant species may become the stronger competitor within the ecosystem, and thus, the parasite could mediate the interaction between the two hosts and alter the outcome of competition (Greenman & Hudson, 2000).

Variation in how parasites affect their different host species has the potential to have community‐wide effects. As our species vary in their susceptibility and tolerance to parasites, the presence or absence of a parasite species within a system may dictate the coexistence of species or the extirpation of a particular species (Begon, Bowers, Kadianakis, & Hodgkinson, 1992; Greenman & Hudson, 2000; Hatcher et al., 2006; Hudson, Dobson, & Lafferty, 2006). The differential effects on amphipods and isopods may lead to community‐wide effects. Understanding the consequences of parasitic infection and differences between host species is key to gaining greater insight into the role of parasite mediation in ecosystem dynamics.

## CONFLICT OF INTEREST

None declared.

## AUTHOR CONTRIBUTIONS

OCF, RP, and CL conceived and designed the experiments. OCF and CL conducted fieldwork. OCF performed the experiments. OCF analyzed the date and wrote the manuscript; other authors provided editorial advice.
